# Epidemiology and Risk Factors for Acute Viral Hepatitis in Bangladesh: An Overview

**DOI:** 10.3390/microorganisms10112266

**Published:** 2022-11-15

**Authors:** Mohammad Enamul Hoque Kayesh, Michinori Kohara, Kyoko Tsukiyama-Kohara

**Affiliations:** 1 Department of Microbiology and Public Health, Faculty of Animal Science and Veterinary Medicine, Patuakhali Science and Technology University, Barishal 8210, Bangladesh; 2 Department of Microbiology and Cell Biology, Tokyo Metropolitan Institute of Medical Science, Tokyo 156-8506, Japan; 3 Transboundary Animal Diseases Centre, Joint Faculty of Veterinary Medicine, Kagoshima University, Kagoshima 890-0065, Japan

**Keywords:** epidemiology, hepatitis A, hepatitis B, hepatitis C, hepatitis E

## Abstract

Viral infections by hepatotropic viruses can cause both acute and chronic infections in the liver, resulting in morbidity and mortality in humans. Hepatotropic viruses, including hepatitis A virus (HAV), hepatitis B virus (HBV), hepatitis C virus (HCV), hepatitis D virus (HDV), and hepatitis E virus (HEV), are the major pathogens that cause acute and chronic infections in humans. Although all of these viruses can cause acute hepatitis in humans, HAV and HEV are the predominant causative agents in Bangladesh, where the occurrence is sporadic throughout the year. In this review, we provide an overview of the epidemiology of hepatotropic viruses that are responsible for acute hepatitis in Bangladesh. Additionally, we focus on the transmission modes of these viruses and the control and prevention of infections.

## 1. Introduction

Hepatitis A virus (HAV), hepatitis B virus (HBV), hepatitis C virus (HCV), hepatitis D virus (HDV), and hepatitis E virus (HEV) are the major causes of hepatitis and are associated with significant morbidity and mortality in developing countries, such as Bangladesh [1]. Acute viral hepatitis causes liver inflammation, which is generally a result of infection with one of the five hepatitis viruses (HAV, HBV, HCV, HDV, and HEV). Acute viral hepatitis may suddenly occur with a usual recovery period of 4–8 weeks. However, HBV, HCV, and HDV can cause chronic infections, resulting in chronic hepatitis, liver cirrhosis, and/or hepatocellular carcinoma [2,3].

Epidemic and sporadic outbreaks of acute hepatitis in low-income countries, such as Bangladesh, are commonly caused by HAV and HEV, which are predominantly transmitted via the fecal–oral route [4]. Notably, HAV is a positive-sense single-stranded non-enveloped RNA virus consisting of a genome of 7.5 kb, encoding a polyprotein, which is posttranslationally cleaved by the virus-encoding proteinase into structural proteins (VP1, VP2, VP3, and a putative VP4) and nonstructural proteins (2A, 2B, 2C, 3A, 3B, 3C, and 3D) [5]. HAV causes 1.4 million infections per year worldwide [6,7]. Further, HBV is an enveloped, circular, and partially double-stranded relaxed circular DNA (rcDNA) virus [8] with four overlapping open reading frames, encoding seven proteins—polymerase, core, precore, three envelope/surface proteins (large, middle, and small), and X protein [9,10,11]. A recent estimate revealed that 296 million people were affected by chronic HBV infection in 2019, with 1.5 million new infections occurring annually [12]. A previous study reported an HBV prevalence of 5.4% in the general population of Bangladesh [13]. HDV is a defective single-stranded RNA virus with a genome of 1.7 kb, requiring HBV as a helper virus for virion assembly and infectivity [14]. The HDV genome has only one ORF, encoding two isoforms of hepatitis delta antigen during replication [15]. HBV/HDV coinfection can cause a more severe course of the disease and an increased mortality compared with HBV single infection [14]. Additionally, HCV is an enveloped, positive-sense single-stranded RNA virus with a genome of ~10 kb and high genetic diversity, resulting in seven major genotypes and more than 60 subtypes [16,17,18,19]. The HCV genome encodes a large polyprotein of approximately 3000 amino acids that is processed by host and viral proteases into three structural (core, E1, and E2) and seven non-structural proteins (p7, NS2, NS3, NS4A, NS4B, NS5A, and NS5B) [17]. According to a recent estimate, approximately 58 million people are currently infected with HCV, with approximately 1.5 million new infections occurring annually [20]. However, the global prevalence of HCV differs considerably among regions [21].

Furthermore, HEV has a single-stranded, positive-sense RNA genome consisting of short 5′ and 3′ non-coding regions (NCRs) and three ORFs (ORFs 1, 2, and 3) [22,23]. This virus has a broad host range, infecting humans, pigs, and birds [23]. To date, eight different genotypes of HEV, HEV1–HEV8, have been identified. Among them, HEV1 and HEV2 are restricted to humans, and HEV3 affects humans, swine, rabbits, deer, and mongooses; HEV4 affects humans and swine; HEV5 and HEV6 are found in wild boars; and HEV7 and HEV8 were recently identified in dromedary and Bactrian camels [23,24]. Notably, HEV is the major cause of acute hepatitis in healthy adults and can cause chronic hepatitis in immunocompromised patients, with increased mortality rates of approximately 25% in pregnant HEV-infected women [25]. Most HEV infections are asymptomatic, and spontaneous viral clearance usually occurs in infected individuals [26]. However, 44,000 deaths due to viral hepatitis caused by HEV were reported in 2015 [27]. Another study that investigated the prevalence of the four common hepatotropic viruses (HBV, HCV, HEV, and HAV) in suspected viral hepatitis patients in Bangladesh collected blood samples from 2995 patients suspected to be infected with HBV, 331 with HCV, 155 with HEV, and 24 with HAV [28]. Serological testing of the collected samples revealed that 245 (8.1%) patients were positive for HBV, 18 (5.4%) for HCV, 87 (56.1%) for HEV, and 8 (33.3%) for HAV infections [28], suggesting that HEV and HAV are highly prevalent hepatotropic viruses in Bangladesh.

Understanding the epidemiology of acute viral hepatitis is crucial to ascertaining the real burden of acute hepatitis. To reduce the spread of the disease, proper understandings of the transmission modes of the viruses (Table 1) and prevention of infection are important. Therefore, in this study, we provide an updated overview of the prevalence of acute viral hepatitis, focusing on infections caused by HAV and HEV, the major pathogens of acute viral hepatitis. We also discuss the transmission modes of hepatotropic viruses and the control and preventive measures to reduce the outbreak of acute viral hepatitis. 

## 2. Acute Hepatitis A

The Hepatitis A virus is considered an important pathogen of acute viral hepatitis. The incubation period for hepatitis A is usually 2–4 weeks [59]. The clinical manifestations of HAV infection vary from asymptomatic infection to acute liver failure (ALF), but do not lead to chronic hepatitis. Hepatitis A causes mild-to-severe manifestations, including fever, malaise, loss of appetite, diarrhea, nausea, abdominal discomfort, dark-colored urine, and jaundice. Clinical manifestations of HAV depend on the age of the patients; mild symptoms are observed in children and severe infections in adults [6]. A recent study reported atypical manifestations of prolonged cholestasis and ascites in 30 (15%) children (<18 years of age) with acute viral hepatitis A in Bangladesh [60]. 

The major risk factors for HAV infection include poor sanitation and hygiene, lack of safe water, living with an infected person, sex with an infected partner, sex between men, and traveling to highly endemic areas without immunization. Owing to the poor sanitary conditions in Bangladesh, most of the population is exposed to HAV during childhood. Notably, the disease is asymptomatic during childhood and provides life-long protection [61]. Well-tolerated and effective vaccines are available to help prevent disease burden and provide long-term protection. A cross-sectional study reported an overall anti-HAV antibody prevalence of 69.6%, increasing with age from 1–5 years (40.4%) to >30 years (98.4%) [62]. Another serological study that investigated 465 participants aged between 1 and 25 years reported a high anti-HAV antibody prevalence (74.8%), suggesting the necessity to screen for HAV antibodies prior to HAV vaccination [63]. Labrique et al. also reported a high prevalence of anti-HAV antibodies (93.5%, 116/124) [64]. A study of 107 patients who were admitted with acute viral hepatitis to the Department of Medicine, Mymensingh Medical College Hospital, Mymensingh, Bangladesh, between April 2017 and September 2017 revealed that 12.15% (13/107) of the patients had acute viral hepatitis A. This study reported mixed HAV and HEV infections at 1.87% (2/107) [1]. However, some studies indicated a high prevalence of HAV infection; in such cases, improved water and sanitation systems are required to reduce transmission rates. A similar pattern was also observed in India, one of the neighboring countries of Bangladesh, where a high seroprevalence (more than 80%) of HAV in children was reported [65,66].

## 3. Acute Hepatitis B

Hepatitis B infection can result in either acute or chronic infection. Healthy adults infected with HBV are usually asymptomatic and can recover without any challenges. However, some people infected with HBV are affected for only a few weeks, exhibiting acute infection, while others may progress to the serious, lifelong illness of chronic hepatitis B. According to an estimate of the global burden of disease, hepatitis B caused 820,000 deaths in 2019 [12]. A previous study reported that, in 2015, approximately 887,000 deaths occurred globally due to acute and chronic HBV infections, with 17%, 40%, and 43% of these deaths occurring due to acute infections, LC, and HCC, respectively [67]. Further, HDV, a satellite virus, can only infect in the presence of HBV, and HDV infection can be acute or chronic. Acute hepatitis D is caused by HBV/HDV co-infection, which refers to simultaneous infection with both HBV and HDV, in an HBV-susceptible individual [68,69]. The combination of HDV and HBV infection is considered the most severe form of chronic viral hepatitis, as it usually accelerates progression toward liver-related death and hepatocellular carcinoma [70]. A previous study suggested that Bangladesh is moderately endemic for HBV infection and indicated relatively high rates of co-infection (24.4%, 44/180) with HDV [71]. Notably, a recent study reported risky sexual behavior (O.R. 4.2; 95% CI: 1.4–12.8) as the sole independent predictor of acute HDV in Italy [72]. 

Several studies have reported a 2–7% HBV carrier rate in Bangladesh [73]. Hepatitis B can be prevented by immunization with safe and effective vaccines. HBV vaccination also provides an effective measure to control acute HDV infection [72]. The World Health Organization (WHO) recommends the hepatitis B vaccine for all newborns, children up to 18 years of age, and all adults at a high risk of infection. Bangladesh introduced the hepatitis B vaccine into the routine childhood vaccination program through the Expanded Program on Immunization (EPI) in 2005, which includes three doses, starting from six weeks after birth [73,74], contributing to the prevention of chronic infections in adulthood. 

In a screening for HBsAg and anti-HBc in 1997 participants between June 2005 and November 2006 in Dhaka, Bangladesh, an intermediate level of endemicity of HBV infection was reported [75]. A seroprevalence study by Labrique et al. reported HBV prevalence of 35.2% (380/1080) [64]. A study of the prevalence of HBV infection in hospitalized children with liver disease in the pediatric department of Mymensingh Medical College Hospital, Bangladesh, between December 2015 and October 2016 reported that 4 of 100 patients had acute hepatitis [76]. Another study in the Department of Medicine, Mymensingh Medical College Hospital, Mymensingh, Bangladesh, between April 2017 and September 2017 reported the prevalence of acute viral B hepatitis as 36.40% (39/107) [1]. A recent meta-analysis reported a 4% prevalence of HBV infection in Bangladesh, which is higher than the global prevalence of HBV infection (3.5%) [77].

Transmission from hepatitis B-infected mothers to children, unsafe blood transfusion by unskilled people, and hazardous use of medical equipment, including syringes, are major risk factors for hepatitis transmission in Bangladesh [78]. Mother-to-child transmission of HCV appears to be the leading cause of pediatric infections [79].

## 4. Acute Hepatitis C

The hepatitis C virus can result in acute or chronic infection. There are limited data on HCV’s prevalence in Bangladesh. However, in a screening of 1997 participants for anti-HCV antibodies between June 2005 and November 2006 in Dhaka, a low prevalence of HCV infection (0.2%, 4/1997) was reported [75]. Another study reported a slightly higher prevalence (0.88%, 9/1018) of HCV infection in Bangladesh, where treatment by unqualified and traditional practitioners, mass vaccination history against smallpox, hair cutting and shaving by barbers, and body piercing were found as major risk factors, rather than the known risk factors of blood transfusion, surgery, invasive therapy, and intravenous drug use [80]. The overall prevalence of HCV in Bangladesh is 0.9%, and G3 is the predominant HCV genotype [19,81]. Although direct-acting antivirals (DAAs) have dramatically improved the outcomes of patients with HCV, new therapeutic options are essential to overcome the limitations of DAAs and improve survival [82,83].

## 5. Acute Hepatitis E

The hepatitis E virus is the most common cause of acute viral hepatitis in the world [84]. Hepatitis E is endemic in many parts of the world, including Central and Southeast Asia, North and West Africa, and Mexico, with disease outbreaks resulting from contaminated food or water. The incubation period following HEV exposure ranges from 2 to 10 weeks, with an average of 5 to 6 weeks [27]. Further, HEV infection is a significant public health issue in many developing countries, including Bangladesh [85]. Although HAV infection remains self-limiting during pregnancy, HEV can cause significant morbidity, with a higher prevalence than that of HAV. Moreover, HEV infection is associated with a very high maternal mortality rate (20%), requiring special attention in endemic areas [86]. A recombinant hepatitis E vaccine, HEV 239, has been approved in China for immunization against HEV infection in adults aged ≥ 16 years [87]. However, the safety and efficacy of the HEV 239 vaccine in certain high-risk populations, such as pregnant women, has not been confirmed [87].

Additionally, HEV is responsible for a wide spectrum of liver diseases, including severe acute viral hepatitis, fulminant hepatic failure, and decompensation of liver cirrhosis [88]. Additionally, HEV is the main cause of acute hepatitis and is estimated to be responsible for 58.33% of acute viral hepatitis cases in Bangladesh [88,89]. In Bangladesh, acute hepatitis sporadically occurs throughout the year, with increased incidence in the rainy season; HEV genotype 1 is prevalent in Bangladesh [90,91,92,93]. According to a WHO report, approximately 2 billion people are infected with HEV each year worldwide, leading to an estimated 3.3 million symptomatic cases of hepatitis E [27]. A previous study confirmed four cases of acute hepatitis E in the fall of 1995 among the United Nations Bangladeshi Peacekeepers in Haiti within a month of deployment to Haiti for peacekeeping duty. The high genomic identity of the strains from these four cases with Asian HEV strains and dissimilarity with the Mexican strain indicated that the strain had indeed been imported from Bangladesh [94]. 

Few studies have been conducted on HEV’s prevalence in the rural Bangladeshi population. One study reported 22.5% (255/1134) seropositivity for anti-HEV IgG in a rural Bangladeshi population, with higher seroprevalence in men (25.8%) than in women (19.7%) [64]. Hossain et al. reported acute viral E hepatitis prevalence of 51.40% (55/107) in a study of 107 patients admitted with acute viral hepatitis to the Department of Medicine, Mymensingh Medical College Hospital, Mymensingh, Bangladesh, between April 2017 and September 2017 [1].

Studies have revealed that HEV infection has multiple risk factors, transmission modes, and zoonotic potentials [95]. Floods are a risk factor for HEV infection, and an increase in acute HEV infections was observed in Bangladesh after the 2004 floods [96]. A retrospective study of 23 patients with fulminant hepatic failure (FHF) revealed that 56.52% (13/23) of them were positive for HEV infection, and 34.78% (8/23) of them were positive for HBV infection [96]. Labrique et al. investigated the HEV infection status in a rural Bangladeshi population and observed 22.5% seroprevalence of anti-HEV antibodies [84].

A previous study reported fulminant hepatitis (FH) patients among an apparently healthy Bangladeshi population, where 63.6% (*n* = 22) of the FH patients were positive for anti-HEV IgM and 7.3% (*n* = 273) were positive for anti-HEV IgM [97], suggesting that HEV infection is highly endemic in Bangladesh. Another study reported that 21.7% (15/69) of HEV infections caused acute hepatitis [91]. In the urban area of Dhaka City, in a study population of 300 adults without any history of jaundice or complaints of liver diseases, HEV prevalence was 30% [98]. 

A previous study investigated HEV seroprevalence in both adults and children by detecting anti-HEV IgG in three hyperendemic areas, including Bangladesh (*n* = 1009), Nepal (*n* = 498), and southwest France (*n* = 1031), revealing that anti-HEV IgG seroprevalence was highest in Bangladesh (49.8%), followed by Nepal (47.1%) and southwest France (34.0%) [99]. Hoa et al. systematically collected serum samples from HEV IgM-positive patients at Bangabandhu Sheikh Mujib Medical University, Dhaka, between August 2013 and June 2015 and found that strains of HEV genotype 1a were dominant in these cases [100]. Additionally, HEV appears to be a cause of adult hospitalization in Bangladesh.

## 6. Comparison of Different Hepatitis Viruses Prevalence 

In a surveillance study, Paul et al. investigated the sera from 1925 patients with acute jaundice who were enrolled in six tertiary hospitals between December 2014 and September 2017 [101]. They reported HEV as a major pathogen of acute hepatitis; 661 (34%) patients had acute hepatitis E, 293 (15%) had acute hepatitis B, and 48 (8%) had hepatitis A [101].

Khan et al. reported the highest HEV prevalence (53%), followed by HAV (39%), HBV (19%), and HCV (13%), suggesting that HEV and HAV are the most common in Bangladesh [102]. Notably, HAV prevalence was very high (100%) among children (≤6 years of age). However, HEV infection was reported to be higher in adults (≥30 years of age) than in children [102]. Another study using sera collected at various hospitals in and around Dhaka from 74 adult patients (aged 15–67 years) indicated concomitant infection in 28 patients with more than one type of hepatitis virus [103]. Notably, 6.75% (5/74) of the cases were positive for HAV, 40.54% (30/74) for HBV, 17.56% for HCV, and 39.18% (29/74) for HEV [103]. Another recent study screened 998 suspected cases of acute hepatitis for anti-HAV IgM and anti-HEV IgM in 10 different hospitals across seven divisions (Dhaka, Chattogram, Rajshahi, Khulna, Sylhet, Barishal, and Rangpur) of Bangladesh and found that 19% (191/998) and 10% (103/998) were positive for HAV and HEV, respectively [4]. 

Notably, a recent study investigated 275 samples obtained from an outbreak of acute jaundice syndrome (AJS) among Rohingya refugees in Cox’s Bazar, Bangladesh, and found that 154 (56%) samples were positive for hepatitis A, 1 (0.4%) for hepatitis E, 36 (13%) for hepatitis B, and 25 (9%) for hepatitis C [104]. Coinfections with multiple etiologies were also reported in 24 samples (9%) [104]. A previous study investigated the etiology of acute hepatitis in hospitalized adult patients (*n* = 165) in Dubai, UAE, between January 2006 and December 2007, and specific etiologic diagnosis of 122 (74%) patients was performed, including acute hepatitis E in 40%, HAV in 18.7%, HBV in 11.5%, HCV in 1.2%, and combined infection in 4.2% of the patients [105]. However, HEV accounted for 54% of acute viral hepatitis cases [105]. In another study conducted in the Department of Hepatology, Bangabandhu Sheikh Mujib Medical University, Dhaka, between November 2003 and May 2008, HEV was reported as the most common cause of FHF and was found in 74.6% (50/67) of patients, followed by HBV infection (4/67) and HAV infection (3/67) [106]; HBV and HAV were found in 5.97% (4/67) and 4.47% (3/67) of the patients with FHF, respectively [106]. Another study reported a seroprevalence of 20% (95% confidence interval (CI), 17–24%) in serum samples from 2924 individuals collected from 70 communities representing all divisions of Bangladesh between October 2015 and January 2016 [107]. As observed, many studies indicated a high prevalence of HEV infection in Bangladesh, drawing attention to the need for improved sanitation and hygiene practices.

## 7. Control and Prevention

Immunization is the primary tool for the prevention of HAV and HBV infections. Despite the availability of an effective vaccine, a low–intermediate prevalence of HBV infection (4%) is present in Bangladesh [77], signifying the necessity of enhanced vaccination. However, HBV vaccination in infancy reduces the risk of adult infection. Notably, infant hepatitis B vaccination in Bangladesh covers over 90%, which should significantly reduce chronic infections, liver cancer, and cirrhotic cases in adulthood [108]. However, conducting surveillance for acute viral hepatitis, including hepatitis A, B, C, and non-ABC hepatitis, to improve disease burden estimates is crucial. In Bangladesh, collaboration between the government public health authority and private laboratories can improve the capacity of outbreak detection and surveillance. Further, HAV is highly contagious and can spread through close personal contact with an infected person or consuming contaminated food or drinks. The availability of an effective preventive vaccine against HAV renders vaccination the best way to prevent hepatitis A. To reduce the risk of HAV and HEV infections, effective control measures should be taken, including drinking pure water, adopting standard sanitation and hygiene practices, and introducing HAV and HEV vaccines to high-risk groups. People can lower the risk of HEV infection by drinking only purified water when visiting countries where hepatitis E is common and by avoiding raw or undercooked pork, venison, and wild boar meat. Notably, good collaboration between human and animal health and the environment should be taken into consideration in the one health concept.

Moreover, a well-tolerated HEV vaccine is available; however, China has only approved the use of HEV vaccines against hepatitis E [109]. In addition, the WHO calls for further studies to confirm the safety and immunogenicity of this vaccine in vulnerable populations and to evaluate its protection in pregnancy [110]. Notably, a phase IV trial was conducted to assess the effectiveness, safety, and immunogenicity of the HEV 239 vaccine (Hecolin) in Bangladesh [110]; however, it is not used in Bangladesh yet. 

According to expert opinion, to prevent HBV transmission from a hepatitis B-infected mother to her child, every pregnant woman should be tested for HBV, and all children should receive the first dose of vaccine within 24 h of birth (birth dose), followed by two doses [78]. Commercial vaccines are not available for hepatitis C infection. 

## 8. Conclusions

In conclusion, HAV and HEV infections are the most common causes of acute hepatitis in Bangladesh. However, the real magnitude of HAV and HEV infections and HAV- and HEV-related acute hepatitis is under-recognized in Bangladesh, requiring further attention. In general, people in Bangladesh remain at a high risk of viral hepatitis due to poor health, inadequate education, poverty, illiteracy, and insufficient hepatitis B vaccination. In addition, the lack of information on the prevalence of hepatitis in the general population is responsible for the high prevalence of the disease. Good hygiene practices are the cornerstone for preventing hepatitis A and E infections. Public awareness should be developed to improve water quality, sanitation, and hygiene practices for controlling HAV- and HEV-related acute hepatitis. Adequate plans are required to strengthen the sanitation programs and vaccination strategies in Bangladesh in order to control and prevent infections. 

## Figures and Tables

**Table 1:** Properties of hepatotropic viruses and their transmission modes.

Characteristics	Hepatitis A Virus (HAV)	Hepatitis B Virus (HBV)	Hepatitis C Virus (HCV)	Hepatitis D Virus (HDV)	Hepatitis E Virus (HEV)
Nucleic acid	RNA	DNA	RNA	RNA	RNA
Genome size	7.5 kb [29]	3.2 kb [30]	10 kb [16]	1.7 kb [31]	7.2 kb [32]
Family	*Picornaviridae*	*Hepadnaviridae*	*Flaviviridae*	*Kolmioviridae* [33]	*Hepeviridae* [34]
Genus	*Hepatovirus* [35]	*Orthohepdnavirus* [36]	*Hepacivirus*	*Deltavirus* [37]	*Orthohepevirus* [38]
Bloodborne transmission	Uncommon	Yes	Yes	Yes	Rare
Foodborne transmission	Yes	No	No	No	Yes
Waterborne transmission	Yes	Not likely	Not likely	Not likely	Yes
Sexual transmission	Not common, may occur	Occur	Not common, may occur	May occur [39]	May occur [40,41,42]
Mother-to-child transmission	May occur, not common [43]	Common [3]	Occur [44]	Possible but rare [45]	Rare [43]
Sharing of needles, syringes, or other drug-preparation equipment	Can facilitate transmission [46]	Constitutes risk factor for hepatitis B [47]	Constitutes risk factor for hepatitis C [48]	Constitutes risk factor for hepatitis D [47]	Not likely [49]
Natural host	Primates [50]	Humans and chimpanzees [51]	Humans	Humans	Humans, pigs, macaque species, chimpanzees, and owl monkeys [52,53]
Animal models for infection study	Chimpanzees, tamarins, and owl monkeys [53]	Woodchucks [51] and tree shrews [54]	Chimpanzees [55] and tree shrews [56]	Chimpanzees and woodchucks [57]	Macaques, chimpanzees, and owl monkeys [53]
Incubation period	2 to 4 weeks	30 to 180 days	14 to 180 days	3 to 25 weeks	2 to 10 weeks
Chronicity	No	Yes	Yes	Yes	Very rare
Liver cancer	No	Yes	Yes	Yes	Very rare [58]
Preventive vaccine	Available	Available	No	No; however, HBV vaccine can protect future hepatitis D infection.	Only available and approved in China

## Data Availability

Not applicable.
